# Vascular Abnormalities Detected with Chest CT in COVID-19: Spectrum, Association with Parenchymal Lesions, Cardiac Changes, and Correlation with Clinical Severity (COVID-CAVA Study)

**DOI:** 10.3390/diagnostics11040606

**Published:** 2021-03-29

**Authors:** Salah D. Qanadli, Alexander W. Sauter, Hatem Alkadhi, Andreas Christe, Pierre-Alexandre Poletti, Lukas Ebner, David C. Rotzinger

**Affiliations:** 1 Department of Diagnostic and Interventional Radiology, Lausanne University Hospital and University of Lausanne, Rue du Bugnon 46, 1011 Lausanne, Switzerland; salah.qanadli@chuv.ch; 2 Department of Radiology, University Hospital Basel, University of Basel, 4031 Basel, Switzerland; alexander.sauter@usb.ch; 3 Institute of Diagnostic and Interventional Radiology, University Hospital Zurich, University of Zurich, 8006 Zurich, Switzerland; Hatem.Alkadhi@usz.ch; 4 Department of Radiology, Division City and County Hospitals, Inselgroup, Bern University Hospital, University of Bern, 3004 Bern, Switzerland; Andreas.Christe@insel.ch; 5 Emergency Radiology Unit, Service of Radiology Division of Clinical Epidemiology Service of Radiology, Geneva University Hospital, 1205 Geneva, Switzerland; Pierre-Alexandre.Poletti@hcuge.ch; 6 Department of Diagnostic, Interventional and Pediatric Radiology, Inselspital, Bern University Hospital, University of Bern, 3010 Bern, Switzerland; Lukas.Ebner@insel.ch

**Keywords:** COVID-19, computed tomography, perfusion, pulmonary embolism, vascular congestion, respiratory failure

## Abstract

Although vascular abnormalities are thought to affect coronavirus disease 2019 (COVID-19) patients’ outcomes, they have not been thoroughly characterized in large series of unselected patients. The Swiss national registry coronavirus-associated vascular abnormalities (CAVA) is a multicentric cohort of patients with severe acute respiratory syndrome coronavirus 2 (SARS-CoV-2) infection who underwent a clinically indicated chest computed tomography (CT) aiming to assess the prevalence, severity, distribution, and prognostic value of vascular and non-vascular-related CT findings. Clinical outcomes, stratified as outpatient treatment, inpatient without mechanical ventilation, inpatient with mechanical ventilation, or death, will be correlated with CT and biological markers. The main objective is to assess the prevalence of cardiovascular abnormalities–including pulmonary embolism (PE), cardiac morphology, and vascular congestion. Secondary objectives include the predictive value of cardiovascular abnormalities in terms of disease severity and fatal outcome and the association of lung inflammation with vascular abnormalities at the segmental level. New quantitative approaches derived from CT imaging are developed and evaluated in this study. Patients with and without vascular abnormalities will be compared, which is supposed to provide insights into the prognostic role and potential impact of such signs on treatment strategy. Results are expected to enable the development of an integrative score combining both clinical data and imaging findings to predict outcomes.

## 1. Introduction

A subset of patients infected with severe acute respiratory syndrome coronavirus 2 (SARS-CoV-2) will develop pneumonia and severe disease [1,2], challenging healthcare providers because the physiopathological mechanisms are unsatisfactorily understood. Hypoxemia leading to mechanical ventilation may be the consequence of several factors, of which thromboembolism is emerging as a key component since blood hypercoagulability is common among hospitalized patients with coronavirus disease 2019 (COVID-19) [3,4,5,6,7].

Since the SARS-CoV-2 outbreak, computed tomography (CT) imaging has almost immediately established itself as the primary non-invasive test for diagnosis, monitoring of COVID-19 pneumonia, and complications thereof, including deep-learning-based analysis [8,9,10,11,12,13,14].

While most of the currently available literature relies on non-contrast CT [10,15], the need to assess vascular abnormalities is being recognized as an increasingly important factor [16,17,18,19], both to help distinguish COVID-19 pneumonia from other viral infections and to exclude pulmonary embolism (PE). Acute PE is believed to be a significant contributory factor in patients with adverse outcomes [3,6,7,20], and anticoagulation therapy was found to reduce mortality in severe COVID-19 disease [21].

Although vascular involvement is thought to aggravate COVID-19 morbidity and mortality, there are still unresolved issues regarding the nature and impact of cardiovascular abnormalities. Furthermore, no convincing theory helps understand the interaction between virus-induced inflammatory disorders and morphologic changes, especially those observed on CT. In addition, the severity of hypoxemia in COVID-19 patients seems to be related to more complex mechanisms than morphologic damages observed in CT [22].

The prevalence of PE in unselected patients is still debated regarding thromboembolic complications. Clot burden/distribution (anatomic distribution, relationship to ground-glass opacity, and clinical severity) is yet to define. Moreover, the association between PE and important clinical variables lack, including time from onset, severity, age of patients, risk factors for venous thromboembolic disease (VTD), and anticoagulation prophylaxis regimen.

Recently vascular changes other than PE have drawn attention [23]. Additional knowledge is, however, required and not yet available to confirm and better understand early observations. In particular, a radiological sign referred to as “vascular thickening”, “vascular enlargement”, or “vascular congestion” (VC) that is thought to be a specific marker of COVID-19 pneumonia calls for a thorough assessment. Quantitative analysis of this sign and correlation to clinical presentation is highly desirable and may help understand its pathophysiology [24].

Most valuable information to address these open issues will likely come from severely ill patients and those who die since a recent autopsy study reports thromboembolism in 50% of people who died from COVID-19, emphasizing the critical role of PE in adverse outcomes [25].

Consequently, we probably underestimate the role of vascular changes and complications induced directly or indirectly by the coronavirus. Seeking a better understanding of the disease is undoubtedly a step toward better managing COVID-19 and its cardiovascular complications.

## 2. Methods and Analysis

### 2.1. Study Design

This study aims to explore COVID-19 features on CT with specific regard to vascular changes.

The data and the conclusions of this study could enhance the clinical care, risk stratification, and, ultimately, clinical outcomes of patients affected with severe COVID-19.

Specifically, this multicenter observational study is designed to comprehensively picture the spectrum of vascular findings related to COVID-19 pneumonia and find correlations with outcomes.

To this end, we will analyze lung parenchymal findings in patients with COVID-19 infection, including their relationship to vascular changes. Vascular abnormalities will be subdivided into PE and non-PE-related lesions. Qualitative interpretation by expert cardiothoracic radiologists and state-of-the-art quantitative analyses will be conducted, including computer-based assessment.

#### 2.1.1. Primary Objective

The study aims to describe the prevalence of vascular abnormalities in COVID-19 pneumonia, especially PE.

#### 2.1.2. Secondary Objectives

Secondary objectives include the frequency of various other factors (as described hereafter) and their potential impact on outcome or treatment.

We will measure the frequency of specific vascular abnormalities related or not to PE and compare their prevalence.

### 2.2. Patient Selection

Patients will be recruited in six Swiss university-affiliated institutions from five cantons (Basel, Bern, Geneva, Vaud, Zurich). For this purpose, each center needs to screen hundreds of COVID-19 patients to select those who meet the inclusion criteria and do not have any exclusion criteria.

#### 2.2.1. Inclusion Criteria

Patients admitted for COVID-19 (with positive reverse transcription polymerase chain reaction for SARS-CoV-2) who had a chest CT within the specified timeframe.

#### 2.2.2. Exclusion Criteria

Age less than 18 years, patients with another pre-existing infectious process, non-optimal CT scan or incomplete CT data, documented refusal of the reuse of medical data.

#### 2.2.3. Sample Size Calculation

The sample size estimate is based on the primary objective of the study. We assume that PE-positive patients have clots in two lung segments on average; the sample size is designed for 80% power and a type-one error rate of 5% [26]. Under the hypothesis that macroscopic PE is related to a systemic hypercoagulability status and does not result from in situ thrombosis due to alveolar inflammation, the probability of an embolus to be located in a segment with vs. without alveolar opacity should be roughly 50-50. To reject this hypothesis and determine a statistically significantly higher incidence of PE in lung segments with alveolar inflammation (20% increase, from 50% to 70%), we need to analyze 182 lung segments with PE. In other words, we need to enroll 91 patients with PE; since the literature reports a PE incidence of 20% in COVID-19 pneumonia [27], we need to enroll at least 500 patients with COVID-19 taking into account a safety margin for excluded patients and those who have declined to participate. By recruiting patients in the most prominent centers in Switzerland, we may reach 1000 patients with COVID-19 pneumonia, of which around 20% will also have PE.

## 3. Methodology and Data Analysis

**Figure 1:**
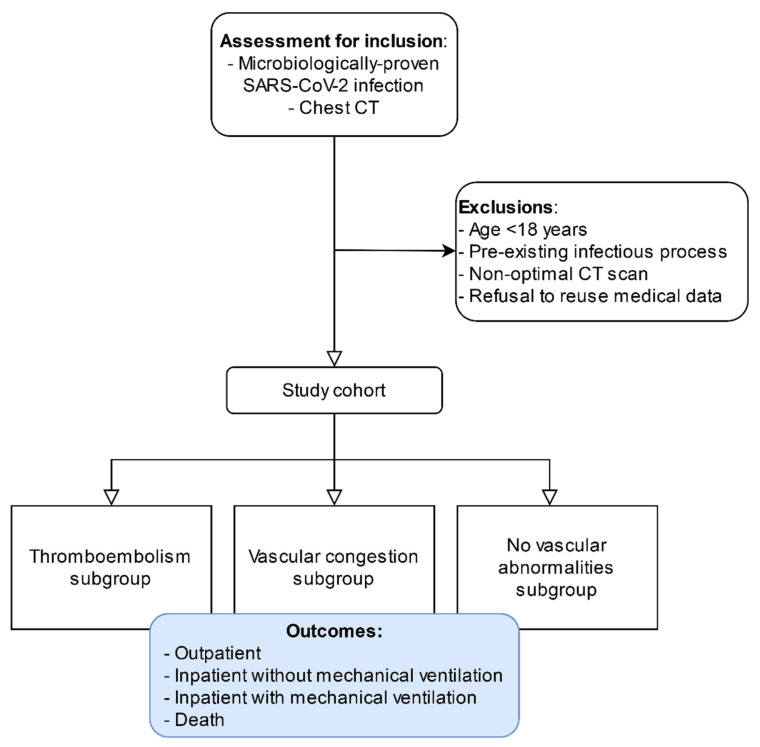
Study flowchart.

We will retrieve clinical, laboratory, and imaging data of eligible patients. Demographic data, including age and sex composition, will be analyzed. Intra-hospital medical records, laboratory test results, and data from chest CT performed in the participating centers will be collected in a Research Electronic Data Capture (REDCap)-based multicenter registry. The study flowchart is reported in Figure 1.

### 3.1. CT Analysis

CT Scans Are Analyzed to Identify:

- PE related abnormalities: the presence of embolic material, anatomic distribution based on segmental arteries, parenchymal changes and their distribution (PE present in the region of interest subject of parenchymal changes induced by the coronavirus), presence of perfusion defect–using iodine vs. water material decomposition if dual-energy CT was performed–assessment of right ventricle, left atrium, and pulmonary artery dimensions (diameters), and quantification of vascular obstruction using the Qanadli obstruction index (QOI) [28,29] and a modified Qanadli obstruction index (mQOI) based on the segmental analysis as follows:

mQOI = (⅀SQOI + ⅀LQOI + ⅀TQOI)/120

where

S: segmental QOI calculated for each segmental artery
L: lobar QOI calculated for each lobar artery
T: troncular QOI calculated for each pulmonary artery

- Non-PE-related vascular abnormalities consist of visual assessment of VC (arterial and venous), manually drawn regions-of-interest in normal and abnormal parenchyma, quantification of vascular volumes and tissue volumes, quantification of venous dilatation, and arterial enlargement.
- Non-vascular abnormalities include ground-glass opacities, consolidation, cysts, nodules, and pleural changes. Semi-quantitative assessment of SARS-CoV-2-related opacities is provided per segment: alveolar opacities (none, <50%, >50%) and per patient. A new relative volume-based index is calculated as follows:

⅀VROI/⅀VL

where

V: volume
ROI: region of interest with parenchymal changes
L: pulmonary lobe

Finally, predictive modeling will be performed to derive an integrative score accounting for both clinical variables and imaging findings to classify the disease severity better and predict patient outcomes.

### 3.2. Data Management

All data will be coded and gathered using REDCap, a Human Research Act (HRA)-compliant electronic data collection platform [30]. REDCap is a secure, web-based platform providing data collection and management in research.

### 3.3. Statistical Analysis

For statistical analysis, we will conduct correlation analysis with Spearman’s rank test, group comparison of qualitative data with Wilcoxon signed-rank test, group comparison of quantitative data with Pearson’s Chi-square test. The inter-observer agreement will be measured by Cohen’s Kappa test for ordinal data and with the intra-class correlation coefficient for continuous data. Outcome modeling will be performed using logistic regression analysis.

Collected data and variables under evaluation are summarized in Table 1 and Table 2. Depending on the evolution of this worldwide pandemic and increasing knowledge concerning new drugs to manage COVID-19 pneumonia, e.g., tamoxifen [31], an amendment might be submitted to the Ethics Committee to analyze additional variables.

## 4. Discussion and Clinical Relevance

While most of the currently available literature relies on non-contrast CT, the need to assess vascular abnormalities is being recognized as an increasingly important factor, both to help distinguish COVID-19 pneumonia from other viral infections and to exclude pulmonary embolism (PE). Acute PE is believed to be a significant contributory factor in patients with adverse outcomes.

The nature of blood clots (arterial thrombosis versus arterial embolism) in the context of COVID-19 is currently debated [32]. Because of this, one of the most critical analyses we will conduct is to assess whether pulmonary blood clots are systematically associated with signs of pneumonia (in the same lung segment) and shed light on the mechanisms underlying vascular changes. Moreover, the association between unfavorable outcomes and alveolar opacities and/or PE will be an essential result for a better insight into the disease course.

Arterial thrombosis in COVID-19 may be due to inflammatory cytokines (suggestive of PE), endothelial dysfunction, or hypoxia (suggestive of local thrombosis). Likewise, VC can be linked to hyperemia in the context of lung inflammation or other factors such as in situ venous thrombosis or vasodilatation triggered by cytokines. A better understanding of these processes would support decision-making, specifically regarding the use and dosing of anticoagulation therapy in severe COVID-19.

Several factors might potentially limit this study. First, some relevant biological markers of cytokine storm, such as interleukin 6 (IL-6), are not routinely collected in all centers and are not expected to be available for analysis. Second, a common pitfall is linked to the enrollment process; since this study only considers patients with microbiologically proven SARS-CoV-2 infection for inclusion, a potentially significant proportion of patients will remain undetected and excluded from the analysis. However, since the endpoints are mainly related to patients with serious or severe disease, the impact should be limited. Finally, should the data fail to offer a predictive value concerning patient outcomes, we may need to review the data and elucidate if potential confounders influence the main effect.

## 5. Ethics and Dissemination

The study protocol was submitted through the Swiss Business Administration System for Ethics Committee (BASEC) and approved by the independent Cantonal ethics committees in charge. All procedures will be conducted by the leading institution (Lausanne University Hospital) and participating institutions (Bern, Zurich, Basel, and Geneva University Hospitals) under the Federal Act on Research involving Human Beings (Human Research Act, HRA). The study will be conducted in compliance with the protocol, the current version of the Declaration of Helsinki, the International Conference on Harmonization Good Clinical Practices (ICH-GCP), and other locally relevant legal and regulatory requirements. Baseline patients characteristics, primary and secondary outcomes will be published in scientific peer-reviewed journals.

## Figures and Tables

**Table 1:** Clinical and laboratory data to be collected.

Variable	Data	Variable Type
Disease severity	Outpatient, inpatient, death	Qualitative
Composite outcome	ICU admission or death	Dichotomic
Cardiovascular comorbidities		
	Hypertension	
	Atrial fibrillation	
	Coronary artery disease	
	Heart failure	
	Peripheral vascular disease	
	Stroke	
	Chronic kidney disease	
	Hemodialysis	
	Diabetes	
	COPD	
	Asthma	
	Cystic fibrosis	
Onset to CT delay	Number of days	Ordinal
Onset to recovery delay	Number of days	Ordinal
Thromboprophylaxis or anticoagulants	Qualitative	Dichotomic
D-dimers	Plasma concentration	Continuous
PaO2	Arterial blood partial pressure	Continuous
SaO2	Venous blood O2 saturation	Continuous
C-reactive protein	Plasma concentration	Continuous
Thrombocytes	Count per microliter	Integer

**Table 2:** Imaging variables under test.

Variable	Data	Variable Type	Segment	Lung	Patient
Left atrium size	2 axes, continuous	Continuous			x
Right ventricle (RV)	Small axis	Continuous			x
Left ventricle (LV)	Small axis	Continuous			x
Pulmonary artery (PA)	Diameter	Continuous			x
Vascular congestion (VC)	Qualitative	Dichotomic	x	x	x
Vascular volume (VV)	Volumetric	Continuous		x	x
Perfusion (PF)	Qualitative, iodine density map	Ordinal (decreased, normal, increased		x	x
Venous-to-artery ratio (VRR)	Diameter ratio	Continuous	x	x	x
Pulmonary embolism (PE)	Qualitative	Dichotomic	x	x	x
Qanadli obstruction index (QOI)	Percentage	Ordinal (0–100% in 2.5% steps)	x	x	x
Modified QOI (mQOI)	Percentage	Ordinal (0–100% in 2.5% steps)	x	x	x
Ground glasses opacities (GGO)	Qualitative	Dichotomic	x	x	x
Alveolar consolidation	Qualitative	Dichotomic	x	x	x
Cyst	Qualitative	Dichotomic	x	x	x
Nodule	Qualitative	Dichotomic	x	x	x
Lung tissue volume (TV)	Volumetric	Continuous		x	x

## Data Availability

The datasets generated and/or analysed during this study are will be available from the corresponding authors on reasonable request.
